# The Nature, Causes, and Treatment of Erysipelas

**Published:** 1842-04

**Authors:** 


ART. V.

1. *A Treatise on the Nature, Causes, and Treatment of Erysipelas.* By THOMAS NUNNELEY, Lecturer on Anatomy, Physiology, and Pathology in the Leeds' School of Medicine, &c.—*London*, (1841?) 8vo, pp. 307.

2. *Article, Erysipelas.* By J. C. DONELLAN, M.D. (*The Cyclopædia of Practical Surgery*, Part X. October, 1841.)

IT is, as Mr. Nunneley observes, no doubt a "singular circumstance," that when distinct and even voluminous works have appeared on almost every disease to which the human body is liable, no separate treatise should have been devoted to a consideration of the Nature, Causes, and Treatment of Erysipelas. Although, however, we have had no distinct monograph on the subject, it has by no means been neglected by medical and surgical writers in various periodical publications and systematic works. Mr. Nunneley is willing to admit that many of these essays are the able productions of able writers; but something he thinks is yet wanting which may be comprised in a work devoted expressly to the disease. It is scarcely necessary we should remark that there is no subject of practical importance upon which there has existed more contrariety of opinion, than upon that of erysipelas, even among the best and most experienced observers. Almost every writer has some peculiar views as to the nature of the disease. But few coincide as to the treatment it requires, and "scarcely any two have agreed as to the precise signification or extent of the word, or what diseases should be included under the term." In proof of this we refer to the various definitions and subdivisions of erysipelas quoted by Mr. Nunneley from different nosological authorities. Some have supposed that erysipelas is a specific disease, having clearly-defined characters, and that the cutaneous affection is merely the effect of a salutary effort of nature to relieve herself of some morbific matter; others, on the contrary, have disregarded the constitutional symptoms, or supposed them to be dependent upon the inflamed condition of the skin. Vogel appears to have been one of the first to discriminate between the local affection and the constitutional disorder; a distinction which probably suggested to Cullen his further separation between erythema and erysipelas; the former of which he places among the phlegmasiæ, the latter among the exanthemata. "Cutaneous inflammations are of two kinds, commonly distinguished by the names phlegmon and erysipelas. Of the latter there are two cases which ought to be distinguished by different appellations. When the disease is an affection of the skin alone, and very little of the whole system, or when the affection of the system is only symptomatical of the external inflammation, I shall give the disease the name of erythema; but when the external inflammation is an exanthema, and symptomatical of an affection of the whole system, I shall then name the disease erysipelas."* How far these distinctions which have been made between erythema and erysipelas are well founded, Mr. Nunneley regards as at least doubtful; and we think his hesitation to admit them is well founded. If, for example, we take the symptoms mentioned as characteristic of erythema,† and compare them with those of erysipelas, we find them so

* First Lines of the Practice of Physic, vol. i.

† Vide Nunneley's Introduction, and also the works of Willan, Bateman, Parr, Good, Rayer, &c.

merging into each other, that the impropriety of the artificial distinction becomes apparent. In many cases the same disorder which by one writer has been treated of as erythema, by another has been considered to be erysipelas. This discrepancy Mr. Nunneley very fairly urges, would in itself be sufficient to excite strong suspicion of the two being identical; and accordingly we find that some few writers have not only maintained the sameness of the two, but have also included disorders of many other parts under the same head. Into the accuracy of this view of the subject Mr. Nunneley enquires at some length. He first succinctly, and for the student instructively, points out the original difference that must be admitted to exist in the constitutions of different persons, by which they are more or less prone to particular diseases, or to disorders of particular organs or tissues; and besides, that when two persons, who are thus constitutionally different, are affected with the same kind of disease, this difference of temperament will induce considerable modifications in the symptoms, and render variation in the treatment necessary.

"Injuries of precisely the same nature, and in the same situation, which in one person shall induce a limited local action,—phlegmon, either without much constitutional disturbance, or if attended by general symptoms, they will be such as are indicative of power, in which depletants are not only borne, but are highly necessary,—shall in another person, or even in the same at another time, induce a local action in which there is considerable tendency to spread, far and wide, diffuse inflammation, or erysipelas, in which the constitutional symptoms are those of great action with little power, and where depletants are not only not indicated but are positively injurious. Farther, that these different states may arise without external injury, in which the local action may be exhibited upon the surface of the body, or be thrown upon an internal membrane, according as there may be some peculiar determinating cause in the part itself, or elsewhere." (p. 21.)

In the opinion of many writers erysipelas is essentially a specific disease, affecting one tissue alone, characterized by the local disorder, which forms the chief symptom, and upon which the constitutional disturbance depends. This view is quite opposed to that which Mr. Nunneley endeavours to establish, in which the character of the local changes is supposed to depend upon the constitutional peculiarity, rather than upon the organization of the part. No one has more strenuously supported the idea of erysipelas being an inflammation of the skin, or of this and the subjacent cellular membrane, than Mr. Lawrence,* and to his paper Mr. Nunneley chiefly refers in endeavouring to refute this opinion. His critical examination of Mr. Lawrence's views appears to us fair and satisfactory. So far from admitting the propriety or accuracy of confining the term erysipelas to an affection of the skin alone, or even of the skin and subcutaneous cellular membrane, Mr. Nunneley would extend its application as a generic term to the following diseases, which he thinks are naturally grouped together: Erythema; erysipelas, in the forms described under the term, whether of the head and face, trunk or extremities, idiopathic or sympathetic; diffuse inflammation of the cellular membrane of Duncan; puerperal fever; diffuse inflammation of the serous membranes; diffuse inflammation of the mucous membrane; "possibly" some forms of arachnitis; diffuse phlebitis, and also this form of inflammation of the

* Med. Chir. Trans., vol. xiv.

absorbents. Mr. Nunneley expresses his gratification at finding that since his treatise was written, the view which he takes of the nature of erysipelas has in most respects been confirmed by so able a writer as Dr. Alison.* Dr. Donellan, on the other hand, in his article on erysipelas, after referring to various pathological opinions, would limit the term as it has been by Lawrence and others.

"In fine," he says, "until a distinctive symptomatology and special anatomical characters shall have been assigned to internal erysipelas; we should avoid rendering the question more complex, and we shall therefore consider the skin to be the exclusive seat of erysipelas." (p. 144.)

We confess that, after an attentive perusal of Mr. Nunneley's remarks upon the different affections just enumerated, and which he would introduce into the family of erysipelas, we cannot refrain from admitting the very strong presumptive evidence, at least, which he affords, of the propriety of giving to the term a wider latitude than has yet been usually conceded to it. We do not perceive, however, that either the "facts or arguments" he has advanced go so far as "to prove the identity of puerperal fever and erysipelas." That the two diseases are frequently co-existent many have proved, and we know from our personal observation. But the frequent co-existence of two diseases would scarcely lead us to infer their "identity." Still, we are quite sensible of the numerous and weighty facts which Mr. Nunneley has adduced upon this interesting point of pathology, and we think it more than probable he would make converts of many to his opinion. For our own parts we hesitate, and cannot go so far as he does upon this subject.

The *causes* of erysipelas, as enumerated by Mr. Nunneley and Dr. Donellan, we do not deem it necessary to dwell upon. Most of our readers are doubtless aware that it is still an unsettled point in the profession, whether erysipelas be a contagious or an infectious disease.

"By many persons," says Mr. Nunneley, "these two terms are used as synonymous expressions, by others not. If by the word contagious be meant that mode of propagation which implies diffusion by personal contact alone, as syphilis or scabies are spread, then probably erysipelas would not rank as a contagious disease; but if it be intended to express that mode of propagation, in which a disease is conveyed by the contamination of the atmosphere, then most assuredly erysipelas is an infectious or contagious disease, if any are so, and we are to believe the evidence of our own senses, and the testimony of numerous witnesses." (p. 143.)

Dr. Donellan's opinions upon this point lead to the same conclusion. "It is impossible not to admit the existence of an erysipelatous constitution of the atmosphere.† . . . . The difference of opinion that exists in England and France with regard to the contagious nature of erysipelas, would tend to prove the influence that climate and habits of life exert in modifying even the nature of disease. Numerous facts, authenticated by indisputable authority, go to prove the contagious nature of erysipelas in Great Britain and Ireland, while equally respectable authorities in France concur in the opposite opinion." (p. 145.) Without

* Dr. Alison on Inflammation. (Library of Practical Medicine, vol. i. p. 85, 1840.)

† During an epidemic erysipelas that prevailed in Paris, 1828, Dupuytren was reduced to the necessity of postponing all operations that were not very pressing, and in the lunatic asylum at Charenton, all external revulsions were obliged to be suspended. These facts are mentioned by Dr. Donellan, and they are good examples of similar ones which have occurred in the public medical institutions of this country.

meaning to assert that erysipelas is always a contagious disease, we think it may be laid down as a safe, and indeed indispensable rule, for a practitioner to act upon the principle that it may be so, especially when several cases occur at the same time. It is especially incumbent upon practitioners of midwifery to abstain from visiting puerperal women shortly after having seen patients with erysipelas. The same caution will apply with equal force and propriety indeed to other diseases, the contagious nature of which may even be suspected. That many risks of the kind to which we allude, more particularly with reference to lying-in women, are daily incurred by practitioners we know; that no mischief results in the majority of instances we admit; but, however frequent the escapes may be, the practitioner is neither morally nor professionally justified in having exposed his patients to any degree of danger, however slight it may be.

Our limits do not permit us to enter into the *symptomatology* of erysipelas, which is described at much length both by Mr. Nunneley and Dr. Donellan. We must observe, however, that several cases have occurred to us which prove that the "vesications, which by some authors are said always to occur, and which by some nosologists, as Willan, Bateman, and Rayer, are made the basis of classification, are by no means a necessary or an invariable accompaniment in mild cases, even when the face is the seat of the attack, though more common here than in other parts of the body." (Nunneley, p. 159.) Again too, from all we know of the disease, and we have had abundant opportunities of watching its tendency during thirty years in public and private practice, we agree with Mr. Nunneley that "under all circumstances there is a decided tendency in this complaint to assume a depressed type." If there are exceptions to this statement, they are rare we think; too rare to disturb the general rule. Mr. Lawrence, on the contrary, "is quite at a loss to discover in this affection those marks of debility which some have so much insisted upon."*

*Erysipelas in Infants.* Of this formidable malady a brief account is given by both our authors. We are sorry to confirm their opinion of its generally fatal character; and we believe with Dr. Donellan, "that all means, rational and irrational, have been generally found ineffectual, and that favorable terminations are more referrible to spontaneous and uncomputed results than attributable to the treatment employed." This form of the disease is very uncommon among children in private families where habits of cleanliness are observed, and where wholesome air and wholesome food are enjoyed. On the contrary, it is not now uncommon, and was formerly much more frequent, especially in its epidemic form, in crowded localities and institutions, where many children are congregated together and exposed to the deleterious influences of bad air, bad food, and uncleanliness. Referring to the works themselves for the diagnosis, prognosis, and post-mortem appearances of erysipelas, we must devote the remainder of our notice to a brief sketch of the opinions given upon the treatment.

GENERAL TREATMENT. Mr. Nunneley briefly refers to the various modes of practice that have been adopted, and dogmatically insisted upon as the best by different writers. He is inclined to fear that "even

* Med. Chir. Trans., vol. xiv., p. 28.

now," the supposition is too widely spread, that any means which have been successfully and judiciously urged in one case of illness, are therefore indiscriminately to be employed in another, which bears the same name, regarding rather the name than the nature of the thing: "for which error the term specific disease is, in part at least, accountable." For our own parts we feel little or no apprehension upon the subject. We do not believe that the practical blunder referred to by Mr. Nunneley is at all "widely spread." Few practitioners, really deserving the name, ever hesitate at the bed-side of the patient to settle the abstract nosological title of the disease for the guidance of their practice, and if errors are committed in the treatment, they very generally, we believe, are attributable to other causes than the "reverence for a name."

Mr. Nunneley lays down three indications in the treatment of erysipelas: 1, to arrest the progress of the disease in its commencement; if this cannot be accomplished, 2, to avert the evils arising from its progress; the disease being subdued, 3, to remove as completely as may be, the effects of it. To those who regard erysipelas as a strictly specific disease, and a true exanthema, the first indication will appear useless because impracticable. By some then it is maintained that erysipelas cannot be arrested in its progress, but that, like measles or smallpox, it always runs an uniform period. It is not denied that the duration of erysipelas may generally be tolerably uniform after the disorder is fully established, but "that in the early stages, if properly treated, it is never arrested," Mr. Nunneley is not prepared to admit. We quite agree with him. We think with him that every practitioner who has had under his care patients who are obnoxious to repeated attacks of erysipelas must have noticed that where the symptoms, local and general, are at first identical, the disorder will at one time assume a formidable shape, and at another terminate in a short time, the cutaneous affection never having advanced beyond a mere blush: these observations we and many others no doubt have frequently made; and we believe that "these different results depend much upon the treatment employed." We have three females under our care who are subject to erysipelas of the face, and we, as well as our patients, feel quite satisfied that to a great extent the severity and duration of the disease are very much controlled by appropriate treatment upon the very first manifestation of the attack. "An emetic followed by a dose of calomel and a brisk purgative, so as effectually to clear out the primæ viæ," are, in Mr. Nunneley's opinion, the most certain means of accomplishing this favorable termination. And here we may appropriately refer to a point which is in our opinion accurately commented upon by Dr. Donellan, namely, the exaggerated apprehensions generally entertained from the sudden disappearance of even slight erysipelatous affections of the face, whether occurring spontaneously or caused by treatment.

"Erysipelas limited to a small portion of the face often disappears in three days, and even less, without any inconvenience; and persons subject to frequent returns of the disease, often get through their last attacks in two or three days without the smallest accident. It is far otherwise, however, when a first and extensive erysipelas suddenly disappears, (metastatic erysipelas,) as the delitescence is then generally the effect, and not the cause as has been said, of some intercurrent visceral inflammation." (Donellan, p. 149.)

The frequency of this metastasis has been no doubt exaggerated, as may be inferred from the opinion of Louis, whose great experience has never furnished him with an instance of the kind. Such too has been the result of our own, no doubt, more limited observation. In the many cases of erysipelas we have witnessed, we have often heard fears of metastasis expressed, but we never knew it happen. In the treatment of erysipelas as of most other diseases, we shall look in vain for "specific" remedies. We must attack symptoms as they arise, and endeavour as far as we can to suppress them. As Mr. Nunneley very truly says, the difficulty is in the selection and application of the precise measures to be used, and the proper time for applying them with the most effect and advantage.

*Venesection.* In the treatment of erysipelas, venesection, as is well known, has been strongly advised by some, and as warmly opposed by others. Mr. Nunneley gives us a brief abstract of the leading "authorities" upon both sides the question. And certain is it that were we to be guided alone by authority and names, it would be difficult to choose between the two exclusive methods of powerful tonics or vigorous bloodletting; for on either side we have a nearly equal weight of talent, experience, and undoubted veracity. The opinions we have formerly* very briefly expressed upon this subject remain unaltered, and Mr. Nunneley agrees with us in the main. And we cannot help thinking that, however perplexing the contrariety of opinions to which we have alluded, may appear to the reading student, much of the difficulty in determining either for or against the use of the lancet, will vanish at the bed-side of the patient, by the exercise of even an average share of that judgment and discrimination which the practitioner must be supposed to possess. Mr. Nunneley's comments upon the subject appear to us sensible, correct, and impartial. In a disease like erysipelas, ranging from the mildest to the severest degrees; attacking the young and plethoric as well as the old and infirm; those of good constitutions, and with unimpaired viscera, in common with the broken-down drunkard and cachectic habit, it is not merely "out of the question," it is absolute and unqualified folly for any man or "authority" to assert that our treatment is to be uniform. "It must be as diversified, and present as many shades of difference as the symptoms which demand its employment." Passing over some remarks in furtherance of this view, which Mr. Nunneley in a professed treatise very properly introduces, but which can only be required by those who have everything to learn, we come to one point upon which we shall rest for a moment.

Dr. Duncan and Mr. Lawrence treat the opinion that patients in large towns support depletion worse than those in the country as ridiculous; the latter asks "at how many miles bleeding becomes safe or proper, and what size or population of a town renders it inadmissible?" Nevertheless Mr. Nunneley conceives that the opinions of Willan, Bateman, and others, are founded upon reason and supported by experience. "There can be no doubt that on the whole, those who constantly reside in large towns are not so liable to high inflammatory action as those who live in the country, and that the latter not only will bear, but also require more antiphlogistic treatment in the same disorders than the former do." That this is a fact of general truth we are quite convinced. On the whole

* Br. and For. Med. Review, vol. V. p. 367.

Mr. Nunneley thinks that erysipelas is not one of those disorders for the cure of which venesection should form a prominent part of the treatment; and if not practised within a few days after the commencement of the attack, ought not, except under very extreme circumstances, to be practised at all; and he believes there are few cases in which the patient does not recover much sooner if general bleeding has not been practised than where it has. In the following sentiment we perfectly agree:

"It is an easy thing for the purpose of producing an immediate effect, or 'knocking the disease on the head,' as it is often termed, to take from a man two, three, or four pounds of blood; but should he survive, the probability is, that he will not for several years, if for ever, be the sound man he was before the shock his system has had inflicted upon it by such heroic proceedings." (p. 220.)

*Tonics and stimulants.* In adults there are few cases of erysiplas in which bark, or any tonic or stimulant, can be proper at the onset of the disease. In infants quinine may be employed at the commencement of the attack with advantage in most cases. Stimulants may more commonly be used with good effects earlier than tonics, and especially stimulants of a diffusible kind, of which ammonia is certainly the best, as it appears to rouse and give energy to the nervous system, without increasing vascular excitement. At a later stage, when the tongue becomes dark and glazed, the pulse weak and feeble, and the strength failing, wine, or perhaps the accustomed beverage of the patient, gin and porter, may rouse the sinking powers of life.

"There is nothing inconsistent in, at the same moment, both abstracting blood locally and administering stimulants or tonics; and in many cases the two may be practised with great advantage; a part may be gorged with blood, while the system is much depressed." (Nunneley, p. 223.)

*Emetics* are not unfrequently productive of salutary effects in those by no means unfrequent forms of the disease where the symptoms indicate disorder of the biliary secretions. In many instances both Mr. Nunneley and Dr. Donellan believe the disease is cut short by their use. In the opinion of the former the tartarised antimony, recommended by Desault, is the worst form of emetic that can be employed, when it is given alone. He prefers oxymel of squill, and ipecacuanha and antimony wines in equal parts.

*Purgatives,* Mr. Nunneley says, "in by far the majority of cases, if properly used, completely obviate the necessity for venesection, especially if they have been preceded by an emetic." (p. 230.) The selection of particular remedies of the class, and the limits to their use are pointed out.

*Mercurials* are clearly indicated by the manifest derangement of the abdominal secretions, and especially those of the liver.

"Not only in the commencement of an attack of erysipelas is a dose of four or six grains of calomel with jalap or one of the neutral salts proper, but it is often of the greatest use to get the system in some slight degree under the influence of mercury. Calomel, in combination with tartrate of antimony and opium, is one of the best and most important means that we possess of keeping the disease under our control, and preventing the destructive sloughing or effusions." (Nunneley, p. 232.)

*Diaphoretics and diurectics* are useful auxiliaries. Mr. Nunneley recommends as the best diaphoretic and diuretic, to allow the patient to drink freely of simple water where there is great heat and thirst; the water being of the same temperature of the apartment. He has never known any metastasis or other untoward accident occur from its employment. Colchicum, digitalis, and antimony too are remedies of importance.

*Narcotics* are approved of by most practitioners. In the early stages the milder, such as hyoscyamus or extract of poppies, with appropriate combinations are safer than opium. At a later period a full dose of opium or of the acetate of morphia at bed-time is of much benefit. And if opiates lessen the pain and irritation, procure sleep, and diminish excitement, they are of decided use and ought to be freely given. In the later stages of the disease, when the strength fails and there is great suppuration with sloughing, opium in large doses at bed-time, often acts like a charm by allaying pain and procuring refreshing sleep and invigorating the stomach.

"In combination with ammonia, opium in very large quantities is, sometimes from the very first, necessary in the treatment of such cases of the cellular variety of erysipelas as have been described by Travers as the effect of poison upon the system, and by Colles and Duncan as diffuse cellular inflammation; and in those most malignant forms of the affection which arise from the poisonous fangs of a snake, with stimulants and calomel, the system will bear excessive quantities, and it affords our only means of salvation." (Nunneley, pp. 241-2.)

Mr. Liston speaks highly of the extract of aconite; its use, he states, is often followed by great abatement of vascular excitement, so that the necessity for bleeding is done away with. It may be given in doses of half a grain.*

*Turpentine* has been strongly advised in the advanced stages of erysipelas, and cases are on record which really seemed desperate, and yet under its use the patients recovered.

"Certain it is," says Mr. Nunneley, "that in some instances where coma has been intense, the pulse sinking, the tongue dry and glazed, and the teeth imbued with sordes, after other remedies had been abandoned in despair, the administration of the oil of turpentine has apparently saved the patient." (p. 244.)

LOCAL TREATMENT. Local means are perhaps of as much importance in the management of erysipelas as those which act through the system. Mr. Nunneley is not favorably inclined to the application of leeches to parts affected by erysipelatous inflammation. He would generally prefer the local abstraction of blood by means of punctures or incisions. Small punctures are the most generally applicable and the most useful. We have often seen this practice extremely beneficial; but we have no experience of either the necessity or the effect of making these punctures by "hundreds or thousands," as recommended by Dr. Bright.† Puncturing is peculiarly applicable in erysipelas of the face, and is more effectual than on the limbs; and when it is adopted freely, from the commencement of the attack, suppuration will never, or very rarely, take place, even in the loose texture of the eyelids.

*Incisions.* Upon the much-contested practice of making incisions upon the inflamed part, and the extent to which they may generally be carried, much instructive information is afforded both by Mr. Nunneley and Dr. Donellan. Mr. Nunneley's comments upon the very long incisions ad-

* Elements of Surgery, 2d Edit. p. 61. † Bright's Reports, vol. ii. p. 97.

vised by Mr. Lawrence and others appear to us judicious. "The hemorrhage from such long incisions is often very alarming; nor can I wholly disconnect the death of some of the patients, whose cases Mr. Lawrence has with much candour related, from the loss of blood occasioned by the incisions." (p. 260.) Mr. Earle relates the case of a young woman "in which an incision was made which extended from an inch below the great trochanter, to within an inch and a half of the ankle."* We cannot even reflect upon such a mode of practice, without some touch of feeling for the patient; and with Mr. Nunneley we should require, before we would countenance its repetition, the strongest proof that so distressing a measure was imperatively necessary. Mr. Lawrence and Mr. C. Hutchinson are decidedly in favour of the incisions being made at the very commencement of the disease, thinking if then made they prevent its increase, and ward off suppuration and sloughing. Mr. Liston and Baron Dupuytren wait until matter is formed. Mr. Nunneley prefers a medium course. He would be satisfied, in the earliest stages, so far as the local abstraction of blood is concerned, with leeches or punctures, with cold evaporating lotions: but if the inflammation still proceed, uncontrolled by these means, he would not wait until suppuration was fully established and the cellular membrane broken down, but at once make two, four, or six incisions, each two or three inches long, in the most prominent places, or where matter and sloughs are most likely to form, down to the fascia. If there be reason to suspect the inflammation has passed beneath the fascia it also should be divided. Of course incisions are not to be made when the inflammation is limited to the skin.

*Cold evaporating lotions* are of considerable benefit in the treatment of erysipelas, Mr. Nunneley says. He would however consult the feelings of the patient, and substitute warm lotions if cold increased the pain or produced a general feeling of chilliness. We scarcely remember an instance in which our patients have not greatly preferred, and have apparently been more relieved by tepid lotions. Mr. Nunneley has no fear of applying cold freely to the whole scalp when it is the seat of the complaint. Nor should we have, from our own experience, provided it was not distressing to the feelings of the patient; still, while Pearson† is decidedly against the use of cold,—and Liston‡ says that "cold lotions may afford temporary relief, but their use is fraught with the utmost danger,"—and Alibert declares "that to apply cold is madness,"—we think that caution would dictate a preference for tepid lotions in most cases of erysipelatous inflammation, whatever may be the part affected.

*Stimulant applications* to the inflamed part are, we believe, of limited utility. They may be applied with advantage where gangrene threatens, and there is great depression. There are still some other local remedies that have been occasionally employed with more or less benefit in erysipelas. The chief are, absorbent powders to the surface; fomentations; and various ointments; mercurial ointment; nitrate of silver; bandages; and tonic spirituous injections. For the detailed account of the use of these various means we must refer to our authors themselves. But we

* Med. and Physical Journal, vol. 57. † Surgery, p. 217. ‡ Elements of Surgery, p. 65.

must not close our review without bestowing a moment's notice on a remedy of recent application to the treatment of erysipelas, viz.:

*Tincture of Iodine.* This, says Mr. Nunneley, "is a remedy which, so far as I know, has not been used in erysipelas except by myself, and those to whom I have mentioned it." This is a statement which certainly demands some explanation on the part of the author, inasmuch as precisely the same treatment, with the same formula, and in the same disease, was recommended by Mr. Davies of Hertford, in a pamphlet published upwards of two years since, and specially devoted to the subject. A review of this work was given in our Eighth Volume, p. 512; and to this notice we refer for the same information as is contained in this part of Mr. Nunneley's work. Either Mr. Davies is one of "those to whom Mr. Nunneley had mentioned" the practice; or Mr. Nunneley has committed a singular oversight, in overlooking a work made so generally known by the notice taken of it by ourselves and our contemporaries.

Mr. Nunneley's treatise is a sensible and well written essay. He may not probably convert all to his opinions as to the propriety of extending the term erysipelas so far as he has done; he has, however, made out a strong case in favour of the views he advocates, and he is entitled to the somewhat rare praise of having questioned and criticised the opinions of others dispassionately and justly. Dr. Donellan's article is necessarily confined within rather narrow limits, but it gives a well condensed abstract of the result of past experience of the nature, varieties, and modes of management of the disease.

---

